# SLC1A3 promotes survival and immune escape of pancreatic adenocarcinoma by regulating the JAK/STAT pathway

**DOI:** 10.1016/j.gendis.2025.101663

**Published:** 2025-05-03

**Authors:** Yihang Liu, Huimin Chang, Xiaobo Wang, Xue Zhao, Yongjun Dang, Ling Zhang, Shuai Wang

**Affiliations:** a Basic Medicine Research and Innovation Center for Novel Target and Therapeutic Intervention, Ministry of Education, College of Pharmacy, Chongqing Medical University, Chongqing 400010, China; b Basic Medicine Research and Innovation Center for Novel Target and Therapeutic Intervention, Ministry of Education, The Second Affiliated Hospital of Chongqing Medical University, Chongqing Medical University, Chongqing 400010, China

Amino acid metabolism is involved in cell survival and growth. However, how amino acids regulate the pancreatic adenocarcinoma (PAAD) tumor process and cellular functions is still unclear.^1^

To ascertain the potential role of solute-carrier (SLC) genes in tumor progression, we aim to identify SLCs that exhibit differential expression between tumor and normal samples. Furthermore, we seek to determine those SLCs that may influence patient survival and those associated with PD-L1 expression across all SLC genes in 34 TCGA cancer types, encompassing both solid tumors and hematologic malignancies (Table S1). We initially identified the differentially expressed SLC genes (DESGs, *p* < 0.05, |log_2_ fold change| ≥ 1) between tumor and normal samples in each cancer type in the TCGA database (Fig. 1A and Table S2). Notably, these DESGs are cancer type-specific, hinting at unique functional roles of SLCs in different cancers. Furthermore, we found that 101 of these DESGs are shared across at least 10 cancer types (Table S3). To identify the SLCs that may function in cancer prognosis, we conducted survival analysis for all SLCs and identified SLCs that relate to patient survival (survival-related SLC genes, denoted as SRSGs, *p* < 0.05, |hazard ratio-1.0| > 0.1) in all cancer types. The hazard ratios of overall survival for significant (*p* < 0.05) SLC genes are shown in Figure S1 and Table S4. As an established target for tumor immunotherapy, PD-L1 has garnered extensive research attention across various domains, including the development of monoclonal antibodies, small molecule inhibitors aimed at protein–protein interaction modulation, and strategies to alter the characteristics of cold and hot tumors. Our objective is to identify which SLCs influence the PD-L1 pathway. To achieve this, we conducted an analysis utilizing the Spearman correlation coefficient (scc) between SLCs and the CD274 gene, which encodes the PD-L1 protein, to pinpoint SLCs that may exhibit a correlation with PD-L1 (Table S5). The SLC genes that demonstrated a significant correlation with PD-L1 (PD-L1-related SLC genes, denoted as PRSGs, |scc| > 0.3 and *p* < 0.05) were found to be specific to certain cancer types (Fig. S2), with 46 SLCs shared by at least 10 cancer types (Table S6). Notably, thymoma exhibited the highest number of PRSGs at 120, whereas esophageal carcinoma had the lowest count with only 8 PRSGs (Table S7). By integrating data from DESGs, SRSGs, and PRSGs across at least two cancer types, we successfully identified a comprehensive list of 198 candidate SLCs (Fig. 1B and Table S8). This comprehensive approach ensures that our candidate list is robust and covers a wide range of potential genetic factors that may influence PD-L1 expression and cancer progression.

**Figure 1:**
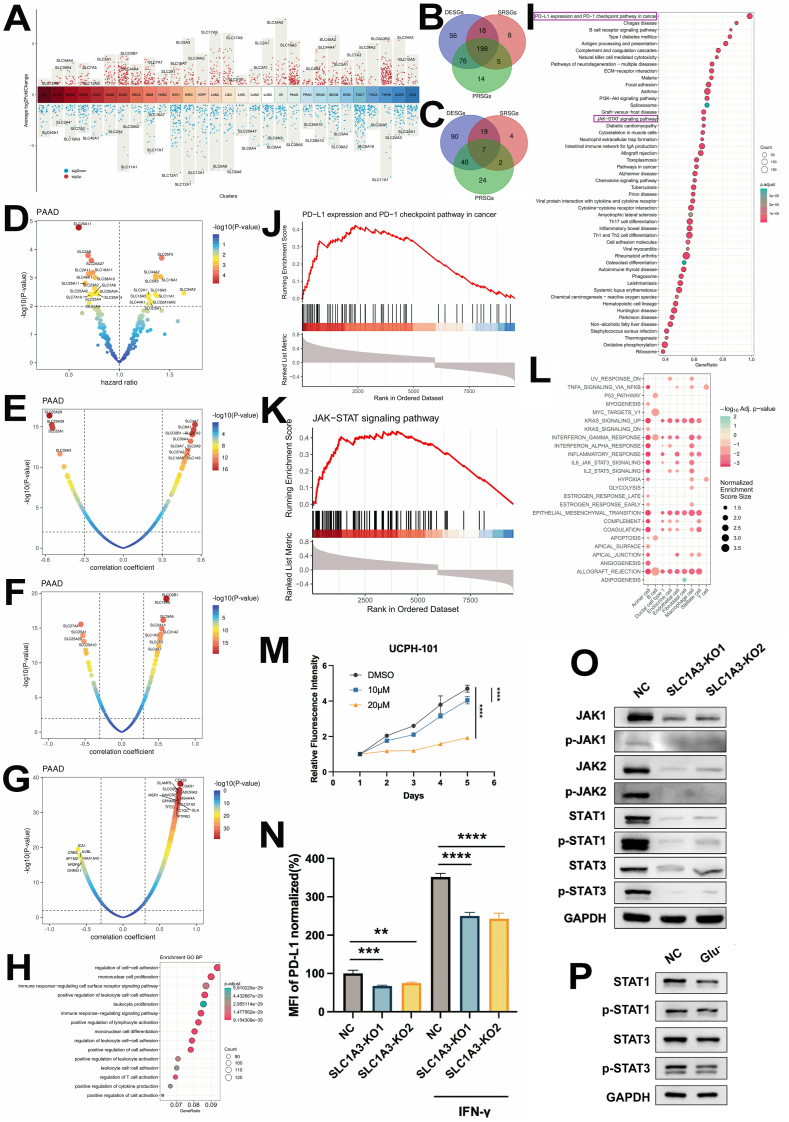
SLC1A3 regulated the JAK/STAT pathway in pancreatic adenocarcinoma (PAAD). **(A)** Differentially expressed SLC genes (DESGs, *p* < 0.05, |log_2_ fold change| ≥ 1.0) in all cancer types. The top one SLC gene was labeled. **(B)** The Venn plot showing the overlap among the three types of SLCs, with DESGs in ≥2 cancer types, PD-L1-related SLC genes (PRSGs) in ≥2 cancer types, and survival-related SLC genes (SRSGs) in ≥2 cancer types. **(C)** Overlap among SRSGs, DESGs, and PRSGs in PAAD. **(D)** SRSGs in PAAD. **(E)** PRSGs in PAAD. **(F)** Correlation between SLC genes and JAK-STAT pathway in PAAD. **(G)** Top genes that are positively and negatively correlated with SLC1A3 in PAAD in TCGA. Immune-related genes are among the top correlated genes. **(H)** Positive similar genes (PSGs) of SLC1A3 in PAAD are enriched in gene ontology biological process terms related to immune response. **(I)** Gene set enrichment analysis (GSEA) using KEGG pathways for similar genes of SLC1A3 in PAAD. **(J)** GSEA results of PD-L1 expression and PD-1 checkpoint pathway in cancer using similar genes of SLC1A3 in PAAD. **(K)** GSEA result of JAK-STAT signaling pathway with similar genes of SLC1A3 in PAAD. **(L)** GSEA enrichment of cancer hallmark pathways for differentially expressed genes between tumor and normal cells in each cell type in PAAD single-cell RNA sequencing dataset. **(M)** The SLC1A3-specific inhibitor UCPH-101 could impair the viability for the wild type. **(N)** The expression of PD-L1 in the total protein was down-regulated in both constitutive and inducible forms via IFN-γ in BxPC3 cell lines knocked out by SLC1A3. **(O)** The JAK/STAT signaling pathway was inhibited in BxPC3 cell lines knocked out by SLC1A3. **(P)** The JAK/STAT signaling pathway was inhibited in glutamate-deficient BxPC3 cell culture. The data were expressed as mean ± standard deviation unless otherwise noted for all figures. *p* values were from two-way repeated measures ANOVA. ∗*p* < 0.05, ∗∗*p* < 0.01, ∗∗∗*p* < 0.001, and ∗∗∗∗*p* < 0.0001.

By overlapping the DESGs, SRSGs, and PRSGs, we identified 7 candidate SLCs in PAAD, including SLC1A3, SLC2A5, and SLC30A1 (Fig. 1C and Table S9). Separately, we initiated our investigation by SRSGs in PAAD (Fig. 1D and Table S10). Among the top SLCs with positive hazard ratios, we found SLC34A2, SLC35F2, and SLC44A2, indicating their potential to be oncogenic. Next, we identified 156 DESGs between tumor and normal samples in PAAD (*p* < 0.05, |log_2_foldchange| > 1) (Fig. S3 and Table S11). These included 138 up-regulated and 18 down-regulated DESGs. To explore the potential for improving immunotherapy in PAAD, we conducted a correlation analysis between SLCs and PD-L1 expression. The three SLC25 family genes, SLC25A29, SLC25A39, and SLC25A1, emerged as the top SLCs negatively correlated with PD-L1 expression, while SLC4A7, SLC8A1, and SLC1A3 showed a positive Spearman correlation coefficient (Fig. 1E and Table S12). Considering that SLCs are primarily involved in solute transport, they might influence the PD-L1 pathway via alterations in ionic concentration and amino acid metabolites within the tumor microenvironment.^2^^,^^3^ Thus, we supposed the possibility might be the JAK/STAT cascade signal, which provides a mechanism for converting extracellular signals into transcriptional responses.^4^ As expected, SLC4A7, SLCO2B1, and SLC1A3 were among the SLCs that positively correlated with both the JAK-STAT pathway and PD-L1 expression (Fig. 1F). In the following analysis, we will focus on SLC1A3, a glutamate transporter, due to its significant correlation with PD-L1 expression and its potential role in cancer progression.

To delve deeper into the function of SLC1A3 in PAAD, we conducted a comprehensive similar gene detection analysis for SLC1A3. Our findings revealed that the expression of 11,132 genes was significantly (*p* < 0.05) correlated with SLC1A3 in PAAD. Among these, 1607 genes exhibited a positive scc (scc >0.4), denoted as positive similar genes, while 364 genes showed a negative scc (scc < −0.4), designated as negative similar genes (Fig. 1G). Gene ontology enrichment analysis further demonstrated that the positive similar genes of SLC1A3 in PAAD were enriched in immunity-related biological processes (Fig. 1H). The negative similar genes of SLC1A3 in PAAD were associated with biological processes such as ATP synthesis and the electron transport chain (Fig. S4). To gain further insights, we performed gene set enrichment analysis (GSEA) on the 11,132 genes whose expression was significantly correlated with SLC1A3 in PAAD using KEGG pathways. The results showed that immune pathways were enriched in this gene set (Fig. 1I). Specifically, PD-L1 expression and PD-1 checkpoint pathway in cancer and the JAK-STAT signaling pathway were all enriched (Fig. 1J, K). These findings provide further evidence for the potential involvement of SLC1A3 in cancer immunity and its regulation of key immune pathways. To further elucidate the intricate relationship between SLCs and immune pathways at the single-cell level, we initially identified differentially expressed genes in each cell type (Fig. S5) and their corresponding hallmark pathways (Fig. 1L) with a single-cell RNA sequencing dataset.^5^ Our analysis revealed that the IL2-STAT5 and IL6-JAK-STAT3 signaling pathways were prominently enriched in acinar cells, macrophages, and endothelial cells (Fig. 1L). Additionally, we examined the DESGs in each cell type (Fig. S6, 7 and Table S13). These findings provide valuable insights into the differential expression patterns of SLC genes across various cell types and their potential implications in immune pathway regulation.

Finally, we confirmed that the findings of the interaction between SLC1A3 and PAAD were centered on the JAK-STAT signaling pathway. The results showed the inhibition of UCPH-101 and the deficiency of SLC1A3 both impaired the viability of PAAD cell line BxPC3 (Fig. 1M; Fig. S8, 9). The flow cytometry results showed that the SLC1A3 KO in BxPC3 could contribute to the reduced expression level of PD-L1 (Fig. 1N; Fig. S10). Figure 1O showed that the JAK-STAT pathway was positively regulated by SLC1A3 in BxPC3. Besides, the deprivation of glutamate and UCPH-101 could impose restrictions on the activity of STAT3 to a certain degree (Fig. 1P; Fig. S11, 12).

Inspired by our findings in solid tumors, specifically PAAD, we sought to assess the situation in hematologic tumors. Intriguingly, we observed that the correlation between SLC1A3 and tumor immunity in acute myeloid leukemia exhibited similarities to that in PAAD. Given that SLC1A3 may play a role in tumor immunity in PAAD, we further investigated its function in a single-cell RNA sequencing dataset of acute myeloid leukemia (Fig. S13 and Table S14, 15). These findings provide new insights into the expression and function of SLCs at the single-cell level in acute myeloid leukemia and suggest potential targets for therapeutic intervention.

Collectively, our study suggests SLC1A3 as a promising immunotherapeutic target for the treatment of PAAD.

## CRediT authorship contribution statement

**Yihang Liu:** Formal analysis, Data curation. **Huimin Chang:** Data curation. **Xiaobo Wang:** Writing – review & editing. **Xue Zhao:** Writing – review & editing. **Yongjun Dang:** Conceptualization. **Ling Zhang:** Data curation. **Shuai Wang:** Conceptualization.

## Funding

This research was funded in part by the 10.13039/501100001809National Natural Science Foundation of China (No. 82204269), the Chongqing Natural Science Foundation (China) (No. CSTB2022NSCQ-BHX0668), and the Science and Technology Research Program of Chongqing Municipal Education Commission (China) (No. KJQN202200427). The computing work in this paper was partly supported by the 10.13039/501100021520Supercomputing Center of 10.13039/501100004374Chongqing Medical University (China).

## Conflict of interests

Yongjun Dang is an Editorial Board member of *Genes & Diseases*. He/she has no involvement in the peer-review of this article and has no access to information regarding its peer-review. The rest authors declare no commercial or financial ties that might be seen as possible conflict of interests during the research.
