# Supplementary material for: SLC1A3 promotes survival and immune escape of pancreatic adenocarcinoma by regulating the JAK/STAT pathway
Source: Genes Dis. 2025 May 3;13(2):101663. doi: 10.1016/j.gendis.2025.101663 (PMC12606991; doi:10.1016/j.gendis.2025.101663)
Supplement: Multimedia component 1 [file mmc1.docx]

**Supporting information**

**The SLC1A3 promotes survival and immune escape of pancreatic adenocarcinoma by regulating JAK/STAT pathway**

**Yihang Liu^1^, Huimin Chang^1^, Xiaobo Wang^1^, Xue Zhao^1^, Yongjun Dang^1,2#^, Ling Zhang^2#^, Shuai Wang^1#^.**

**1 Basic Medicine Research and Innovation Center for Novel Target and Therapeutic Intervention, Ministry of Education, College of Pharmacy, Chongqing Medical University, 400010, Chongqing, China**

**2 Basic Medicine Research and Innovation Center for Novel Target and Therapeutic Intervention, Ministry of Education, the Second Affiliated Hospital of Chongqing Medical University, Chongqing Medical University, 400010, Chongqing, China**

**#Corresponding author:**

**E-mail addresses: shuaiwang@cqmu.edu.cn**

**E-mail addresses: lingzhang02@hospital.cqmu.edu.cn**

**E-mail addresses: yjdang@cqmu.edu.cn**

**Additional Background**

Pancreatic adenocarcinoma (PAAD) is a fatal disease with a poor prognosis and an increasing incidence. Despite significant progress in surgery and multi-drug chemotherapy for cancer treatment, its 5-year survival rate remains only 13%, the lowest among all types of cancers.^1,2^ So far, there is an extreme lack of therapy for advanced pancreatic cancer, and currently approved drugs have limited clinical benefits.^3,4^ Therefore, the urgent priority necessitates the discovery of specific targets for pancreatic cancer and the development of novel targeted therapeutic candidate drugs.

The tumor microenvironment (TME) is complex and dynamic, with cancer cells coexisting with multiple immune cells, cancer-associated fibroblasts, and endothelial cells, often perfused by a poorly formed vascular system.^5^ Recent studies have revealed that among various modes of crosstalk within TME, metabolic reprogramming is a well-recognized hallmark of cancer, driving tumor proliferation by enhancing the ability to acquire nutrients in a complex and often nutrient-limited environment.^6,7^

The transport of materials across membranes plays a key role in metabolism.^8^ Protein transporters are the molecular channels that control this movement and serve as critical regulatory points for these processes, thus making them an attractive class of therapeutic targets.^9^ With more than 400 members, the solute carriers (SLCs) membrane transport proteins are the largest family of transporters, frequently upregulated in cancers to satisfy high nutritional and energetic needs.^10^ SLCs are integral membrane proteins that function as facilitative or secondary active transporters. Essentially, these transporter proteins control the extracellular and cytosolic concentrations of solutes, ultimately influencing cellular metabolism, signaling, and physiology.^11^ Recent studies have revealed that ten SLC transporters have emerged within pancreatic cancer as pivotal factors in chemoresistance, tumor proliferation, and, remarkably, suppression of tumorigenesis.^12^ Additionally, increasing evidence has shown that SLC transporters have emerged as central players in tumor immunotherapy while blocking certain SLCs in the tumor cells can augment immunotherapy. ^13^

SLC transporters are recognized as a class of druggable proteins, yet they remain pharmacologically underexplored, with only a handful of approved drugs targeting them to date.^14^ Developing drug molecules with durable efficacy and favorable biosafety that target SLC transporters thus represents a promising alternative therapy to treat PAAD. Hence, in this study, we screened the SLC superfamily for those significantly related to PAAD and identified the excitatory amino acid transporter 1 (SLC1A3) as a molecular determinant involved in PAAD progression and investigated that SLC1A3 alters glutamic acid metabolism in the tumor microenvironment (TME) and promotes immune evasion by activating the JAK/STAT signaling pathway to upregulate PD-L1. Our research provides a novel drug target and therapeutic strategy for the treatment of pancreatic cancer.

**Materials and Methods**

**TCGA data**

The gene expression RNA-seq IlluminaHiSeq data of TCGA cancer types were download from UCSC Xena browser in log2(norm_count+1) form ([https://tcga-xena-hub.s3.us-east-1.amazonaws.com/download/TCGA.${cancertype}.sampleMap%2FHiSeqV2.gz](https://tcga-xena-hub.s3.us-east-1.amazonaws.com/download/TCGA.$%7bcancertype%7d.sampleMap%2FHiSeqV2.gz)).

This study included 34 cancer types, including ACC, BLCA, BRCA, CESC, CHOL, COAD, DLBC, ESCA, GBM, HNSC, KICH, KIRC, KIRP, LAML, LGG, LIHC, LUAD, LUNG, LUSC, MESO, OV, PAAD, PCPG, PRAD, READ, SARC, SKCM, STAD, TGCT, THCA, THYM, UCEC, UCS, UVM. The full names of the cancer types can be found in Table S1.

SLCs genes are genes whose symbol starts with “SLC”.

**Differentially expressed SLC genes (DESGs)**

Differentially expressed genes (DEGs) between tumor and normal samples of all cancer types in TCGA were downloaded from gepia2 browser (<http://gepia2.cancer-pku.cn>) ^15^with |log2FC| cutoff =0.0 and q-value cutoff=1.0. Differentially expressed SLC genes (DESGs) are SLC genes with p<0.05, |log2FC|>=1.

**Survival analysis and survival related SLC genes (SRSGs)**

Overall survival (OS) of SLCs in each cancer type was calculated by R package ezcox (<https://arxiv.org/abs/2110.14232>). Briefly, the dataset “tcga_RSEM_gene_tpm” were obtained from host <https://toil.xenahubs.net> via fetch_dense_values function in UCSCXenaTools and UCSCXenaShiny R package. Then the expression data was converted to dataframe and scaled and subjected to ezcox_group function with parameters status=”OS”, grp_var = "type", covariate = SLCgene, format_options = forestmodel::forest_model_format_options(point_size = 2)). SLC genes that significantly affect overall survival time (survival related SLC genes, denoted as SRSGs) are those that with p value<0.05, |hazard ratio-1.0|>0.1.

**Correlation analysis and PD-L1 related SLC genes (PRSGs)**

Spearman correlation coefficient (scc) and p value between SLC genes and CD274 genes and JAK-STAT pathway is calculated by scipy.stats.spearman function from scipy python package. PD-L1 related SLC genes (PRSGs) are defined as the SLCs whose scc to CD274 gene fulfills criteria |scc|>0.3 and p-value <0.05. For each cancer type, the expression of JAK-STAT pathway is defined as the mean expression level of all 155 genes involved in this pathway (Table S16). The expression is the norm_count.

**Similar gene analysis**

For each cancer type, similar genes of SLC1A3 are those genes that are significantly correlated to SLC1A3 (|SCC| >0.4 and p value <0.05). Among all similar genes, positive similar genes (PSGs) and negative similar genes (NSGs) are those with scc>0.4 and scc<-0.4, respectively.

**Enrichment analysis**

The enriched GO terms and KEGG pathways were calculated by enrichGO and enrichKEGG function in clusterProfiler R package（<https://bioconductor.org/packages/release/bioc/html/clusterProfiler.html>）^16^. GSEA analysis were conducted by GSEA and gseKEGG function in clusterProfiler package.

**scRNA-seq dataset of PAAD**

The scRNA-seq of Pancreatic ductal adenocarcinoma (PDAC) samples were downloaded under accession number CRA001160 from GSA (Genome Sequence Archive) (<https://download.cncb.ac.cn/gsa/CRA001160>)^17^. Seurat R package was used to visualize the expression of SLC genes. Differentially expressed genes (DEGs) between tumor and normal samples in each cell types were calculated by find_diff_genes function in Scillus package（<https://github.com/xmc811/Scillus>） and visualized by jjvolcano function in scRNAtoolVis package（<https://github.com/junjunlab/scRNAtoolVis>）. Enriched cancer hallmark pathways of significantly differentially expressed genes (p<0.05) in each cell types were analyzed and visualized by test_GSEA and plot_GSEA function in Scillus package, respectively.

**scRNA-seq dataset of LAML**

The Seq-Well single-cell RNA-sequencing (scRNA-seq) dataset of bone marrow aspirate samples from AML patients and healthy control donors were downloaded from GEO under accession number GSE116256. The digital expression matrices of each sample and cell type annotation results were used to downstream analysis. Seurat R package was used to normalize and scale the gene expression. Annotated cells include hematopoietic stem cell (HSC), progenitor (Prog), granulocyte-macrophage progenitor (GMP), promonocyte (Promono), monocyte (Mono), conventional dendritic cell (cDC), plasmacytoid dendritic cell (pDC), early erythroid progenitor (early Ery), late erythroid progenitor (late Ery), progenitor B cell (Pro B), mature B cell (B), plasma cell (Plasma), naïve T cell (T), cytotoxic T Lymphocyte (CTL) and natural Killer cell (NK). Differentially expressed genes between malignant and normal cells in cDC, GMP, HSC, Mon, Prog and proMono were calculated by find_diff_genes function in Scillus package and visualized by jjvolcano function in scRNAtoolVis package. Enriched cancer hallmark pathways of significantly differentially expressed genes (p_adj<0.05) in each cell types were analyzed and visualized by test_GESA and plot_GSEA function in scRNAtoolVis package.

**Cell lines**

Human pancreatic adenocarcinoma cells (BxPC3) were grown in RPMI 1640 Medium (C11875500BT, Gibco, Thermo Fisher) with 10% fetal bovine serum (FBS, Gibco), supplemented with 100 U/ml penicillin G, 100 mg/ml streptomycin sulfate (Pen-strep, Gibco), and cells were maintained at 37 ℃ under 5% CO2.

**Generation of CRISPR/Cas9 knockout BxPC3 lines**

The sgRNA expression plasmid was constructed by phosphorylating and annealing each pair of complementary oligonucleotides that targeted a specific gene using T4 PNK (M0201S, New England Biolabs), then ligating the resulting double-stranded DNA product into a BsmBI-digested lentiCRISPR V2 plasmid backbone using T4 Quick Ligase (Thermo Fisher). The complementary oligonucleotides for each target gene were:

| Gene | Primer-Forward (5ʹ-3ʹ) | Primer-Reverse (5ʹ-3ʹ) |
| --- | --- | --- |
| SLC1A3-KO1 | CACCGTGCGTTTACGGACTCCCTGC | AAACGCAGGGAGTCCGTAAACGCAC |
| SLC1A3-KO2 | CACCGCACAAAAGCATTCCGAAAC | AAACGTTTCGGAATGCTTTTGTGC |

Viral material was packaged by transfecting packaging plasmids PAX2, PGMD2G, and sgRNA expression plasmids with a specific target to each gene to 293T cells. After 8 hours, the medium was replaced with DMEM containing 10% FBS. The supernatant was collected after transfecting for 48 hours and 72 hours. The two supernatants were then mixed, lightly centrifuged, and filtered using a 0.45-μm filter. The viruses were then added to the target cells with 1 μg/ml polybrene. After 48 hours, the medium was completely replaced with medium containing 1 μg/ml puromycin. After 72 hours of puromycin selection, the resistant cells were harvested as knockdown cells. For the knockout cells, puromycin-resistant cells were diluted for colony formation. After the single colony had grown to an appropriate size, the efficacy of knockout was confirmed by western blotting.

**Cell proliferation assay**

Cell proliferation was assayed by CellTiter-Glo Reagent (Promega, G7573), which is an assay that assesses cell viability by indicating the amount of ATP present in a cell culture well. For these experiments, cells were seeded in 96-well microplates at a density of 2,000 cells in 100µl of complete cell culture medium per well and allowed to adhere overnight. 100 µl of CellTiter-Glo reagent per well was added and plates were incubated at room temperature for 10 min. Luminescence was read in an EnVision Multimode Plate Reader (PerkinElmer).

**Chemical agents**

2-amino-5,6,7,8-tetrahydro-4-(4-methoxyphenyl)-7-(naphthalen-1-yl)-5-oxo-4H-chromene-3-carbonitrile (UCPH-101) were purchased from TargetMol.

**Antibodies used in immunoblotting or flow cytometry**

Anti-SLC1A3(20785-1-AP; Proteintech); Anti-Jak1(50996, Cell Signaling Technology);Anti-p-Jak1(74129, Cell Signaling Technology); Anti-Jak2(3230, Cell Signaling Technology); Anti-p-JAK2(8082, Cell Signaling Technology); Anti-STAT1(14994, Cell Signaling Technology); Anti-p-STAT1(9167, Cell Signaling Technology); Anti-STAT3(9139, Cell Signaling Technology); Anti-p-STAT3(9145, Cell Signaling Technology); Anti-human PD-L1 for flow cytometry(329708, Biolegend) or western blotting(A19135, ABclonal) ; Anti-GAPDH (60004-1-Ig, Proteintech). Secondary antibodies conjugated with HRP were purchased from Jackson Immuno-Research.

**Immunoblot**

Cells were seeded in six-well plates and treated as described in the figure legends with the addition of SDS loading buffer (6x) and boiled at 100°C for 10 minutes. Cell lysates were subjected to SDS-PAGE and transferred to a nitrocellulose membrane (66485, Pall). The membrane was blocked with 5% non-fat milk at room temperature for 1 hour after protein transfer. After incubation with the primary antibody overnight, the membrane was washed with PBS-T and incubated with HRP-conjugated antibody in 5% non-fat milk for 1 hour at room temperature. Finally, ECL substrate was used for exposure.

**Flow Cytometry**

Cells were suspended in Stain Buffer (554657, BD Pharmingen) and incubated with primary conjugated antibodies at room temperature for 30 minutes. After washing in Stain Buffer, samples were analyzed with a BD LSRFortessa cell analyzer (BD Bioscience, USA).

**Statistical analysis**

Data analysis was performed using GraphPad Prism 10.0 (GraphPad Software). Statistical analysis was performed using an unpaired t test, One-way ANOVA, or Two-way ANOVA. P values of less than 0.05 were considered statistically significant.

**Reference**

1. Siegel RL, Giaquinto AN, Jemal A. Cancer statistics, 2024. *CA: A Cancer Journal for Clinicians*. 2024;74(1):12-49. doi:<https://doi.org/10.3322/caac.21820>

2. Cai J, Chen H, Lu M, et al. Advances in the epidemiology of pancreatic cancer: Trends, risk factors, screening, and prognosis. *Cancer Lett*. Nov 1 2021;520:1-11. doi:10.1016/j.canlet.2021.06.027

3. Hidalgo M. Pancreatic cancer. *N Engl J Med*. Apr 29 2010;362(17):1605-17. doi:10.1056/NEJMra0901557

4. Ryan DP, Hong TS, Bardeesy N. Pancreatic adenocarcinoma. *N Engl J Med*. Sep 11 2014;371(11):1039-49. doi:10.1056/NEJMra1404198

5. de Visser KE, Joyce JA. The evolving tumor microenvironment: From cancer initiation to metastatic outgrowth. *Cancer Cell*. Mar 13 2023;41(3):374-403. doi:10.1016/j.ccell.2023.02.016

6. Faubert B, Solmonson A, DeBerardinis RJ. Metabolic reprogramming and cancer progression. *Science*. Apr 10 2020;368(6487)doi:10.1126/science.aaw5473

7. Pavlova NN, Thompson CB. The Emerging Hallmarks of Cancer Metabolism. *Cell Metab*. Jan 12 2016;23(1):27-47. doi:10.1016/j.cmet.2015.12.006

8. Giacomini KM, Huang SM, Tweedie DJ, et al. Membrane transporters in drug development. *Nat Rev Drug Discov*. Mar 2010;9(3):215-36. doi:10.1038/nrd3028

9. Wang WW, Gallo L, Jadhav A, Hawkins R, Parker CG. The Druggability of Solute Carriers. *J Med Chem*. 2020/04/23 2020;63(8):3834-3867. doi:10.1021/acs.jmedchem.9b01237

10. Nwosu ZC, Song MG, di Magliano MP, Lyssiotis CA, Kim SE. Nutrient transporters: connecting cancer metabolism to therapeutic opportunities. *Oncogene*. 2023/03/01 2023;42(10):711-724. doi:10.1038/s41388-023-02593-x

11. Wright NJ, Lee SY. Recent advances on the inhibition of human solute carriers: Therapeutic implications and mechanistic insights. *Curr Opin Struct Biol*. Jun 2022;74:102378. doi:10.1016/j.sbi.2022.102378

12. Bharadwaj R, Jaiswal S, Velarde de la Cruz EE, Thakare RP. Targeting Solute Carrier Transporters (SLCs) as a Therapeutic Target in Different Cancers. *Diseases*. Mar 21 2024;12(3)doi:10.3390/diseases12030063

13. Chen R, Chen L. Solute carrier transporters: emerging central players in tumour immunotherapy. *Trends Cell Biol*. Mar 2022;32(3):186-201. doi:10.1016/j.tcb.2021.08.002

14. Dvorak V, Superti-Furga G. Structural and functional annotation of solute carrier transporters: implication for drug discovery. *Expert Opinion on Drug Discovery*. 2023/10/03 2023;18(10):1099-1115. doi:10.1080/17460441.2023.2244760

15. Tang Z, Kang B, Li C, Chen T, Zhang Z. GEPIA2: an enhanced web server for large-scale expression profiling and interactive analysis. *Nucleic Acids Res*. Jul 2 2019;47(W1):W556-w560. doi:10.1093/nar/gkz430

16. Yu G, Wang LG, Han Y, He QY. clusterProfiler: an R package for comparing biological themes among gene clusters. *Omics*. May 2012;16(5):284-7. doi:10.1089/omi.2011.0118

17. Peng J, Sun BF, Chen CY, et al. Single-cell RNA-seq highlights intra-tumoral heterogeneity and malignant progression in pancreatic ductal adenocarcinoma. *Cell Res*. Sep 2019;29(9):725-738. doi:10.1038/s41422-019-0195-y
