# Supplementary material for: SLC1A3 promotes survival and immune escape of pancreatic adenocarcinoma by regulating the JAK/STAT pathway
Source: Genes Dis. 2025 May 3;13(2):101663. doi: 10.1016/j.gendis.2025.101663 (PMC12606991; doi:10.1016/j.gendis.2025.101663)
Supplement: Multimedia component 2 [file mmc2.docx]

# Supplemental figures

# The SLC1A3 promotes survival and immune escape of pancreatic adenocarcinoma by regulating JAK/STAT pathway

**Yihang Liu^1^, Huimin Chang^1^, Xiaobo Wang^1^, Xue Zhao^1^, Yongjun Dang^1,2#^, Ling Zhang^2#^, Shuai Wang^1#^.**

**1** Basic Medicine Research and Innovation Center for Novel Target and Therapeutic Intervention, Ministry of Education, College of Pharmacy, Chongqing Medical University, 400010, Chongqing, China

**2** Basic Medicine Research and Innovation Center for Novel Target and Therapeutic Intervention, Ministry of Education, the Second Affiliated Hospital of Chongqing Medical University, Chongqing Medical University, 400010, Chongqing, China

**#Corresponding author:**

E-mail addresses: shuaiwang@cqmu.edu.cn

E-mail addresses: lingzhang02@hospital.cqmu.edu.cn

E-mail addresses: yjdang@cqmu.edu.cn


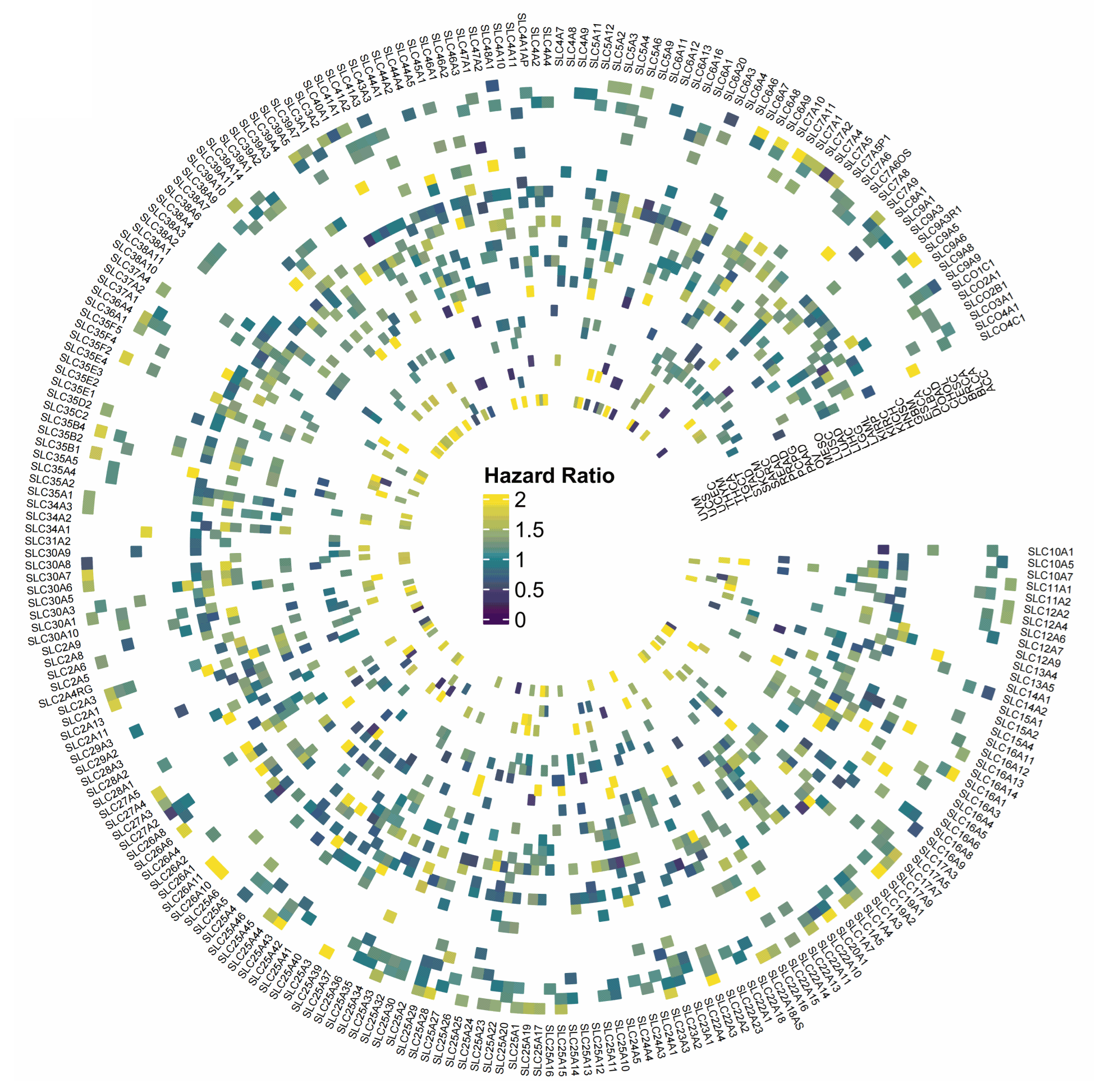


Fig S1. SLC genes that significantly (p value <0.05) affect the overall survival (OS) of patients in the TCGA cancer types. SLC genes with p value >=0.05 were colored in white.


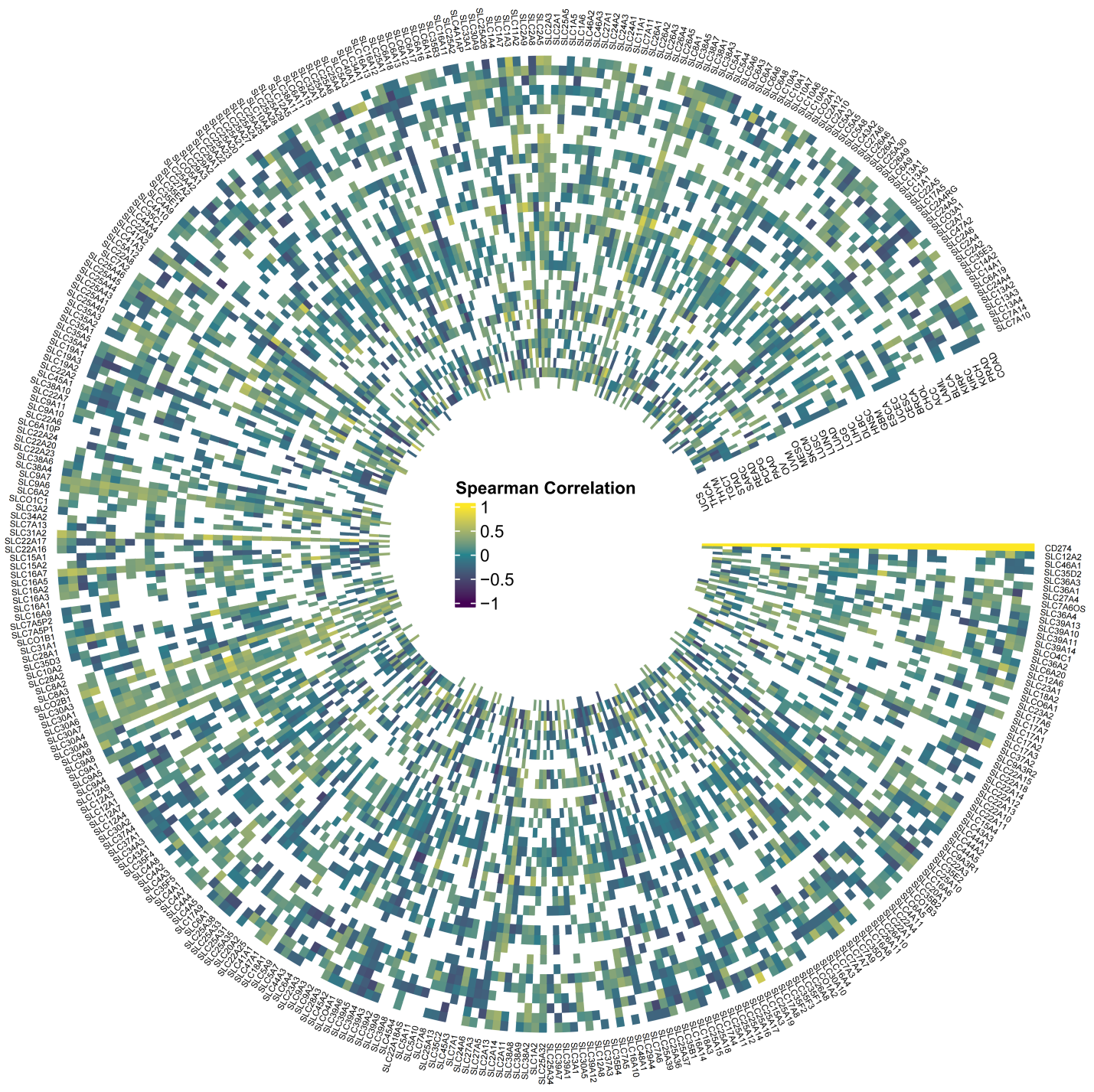


Fig S2. Spearman correlation to CD274 gene of SLC genes in all cancer types. Non-significant correlation (p>0.05) was indicated as white.


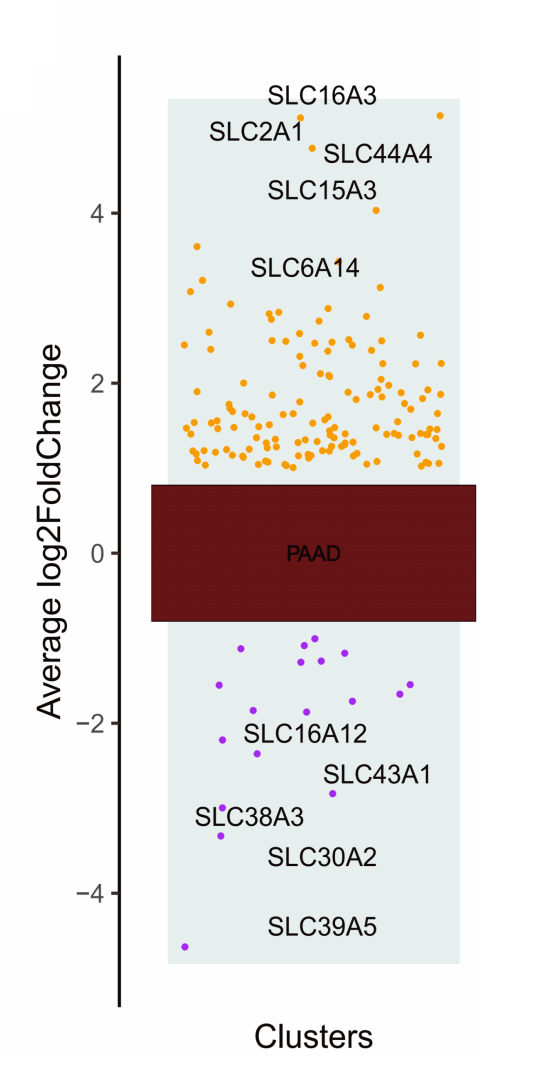


Fig S3. DESGs in PAAD.


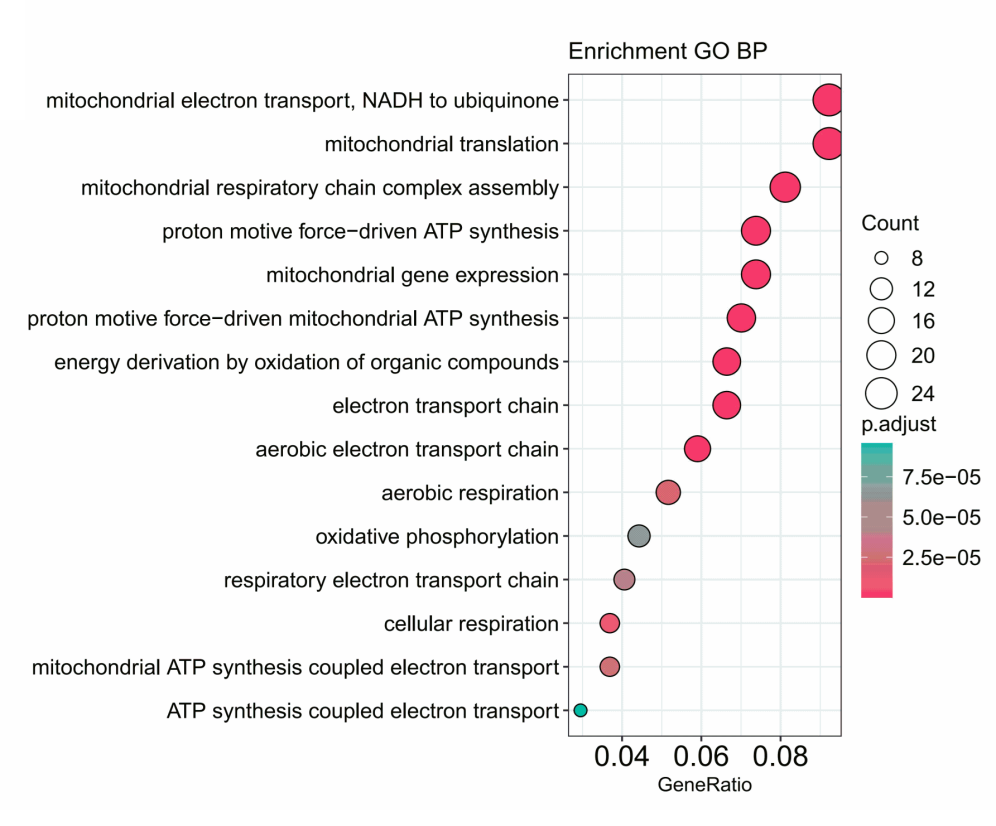


Fig S4. NSGs of SLC1A3 in PAAD are enriched in GO BP terms related to electron transport and ATP synthesis.


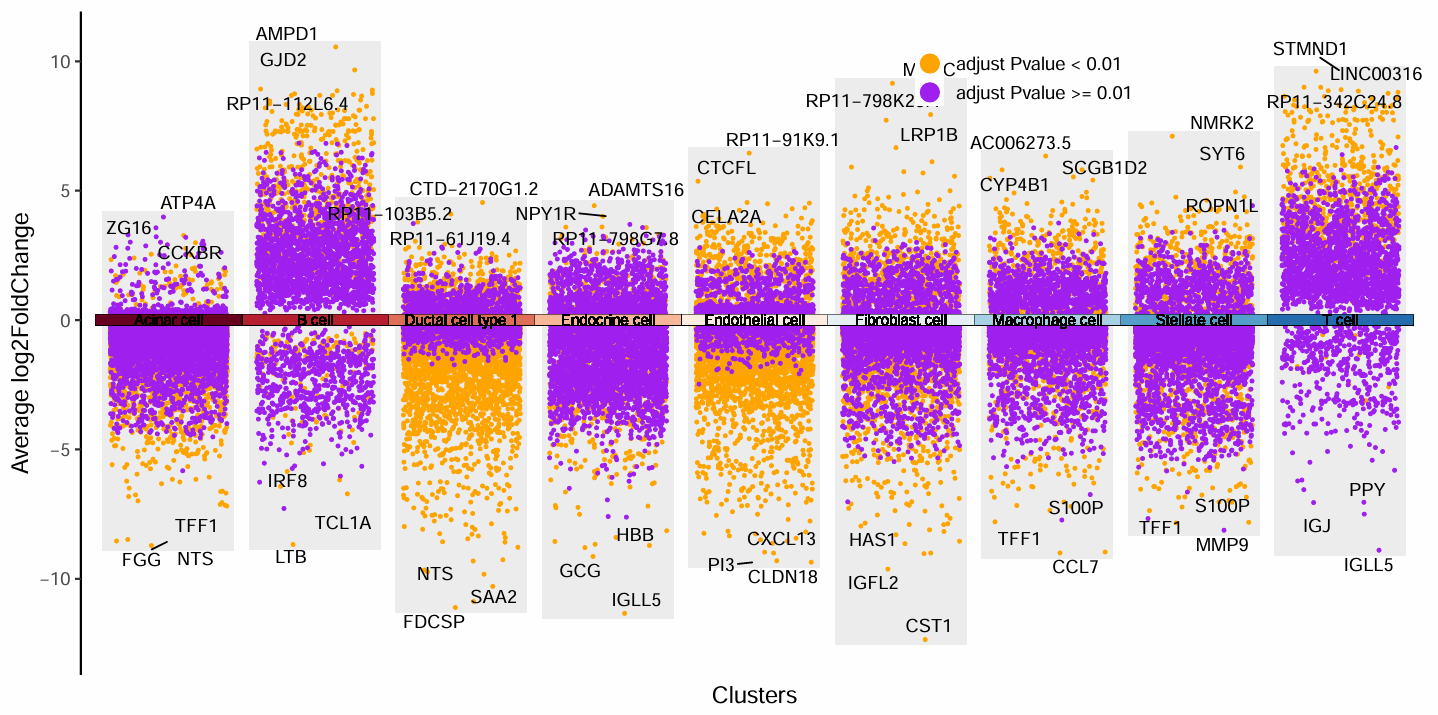


Fig S5. Differentially expressed genes between tumor and normal samples in PAAD scRNA-seq dataset.


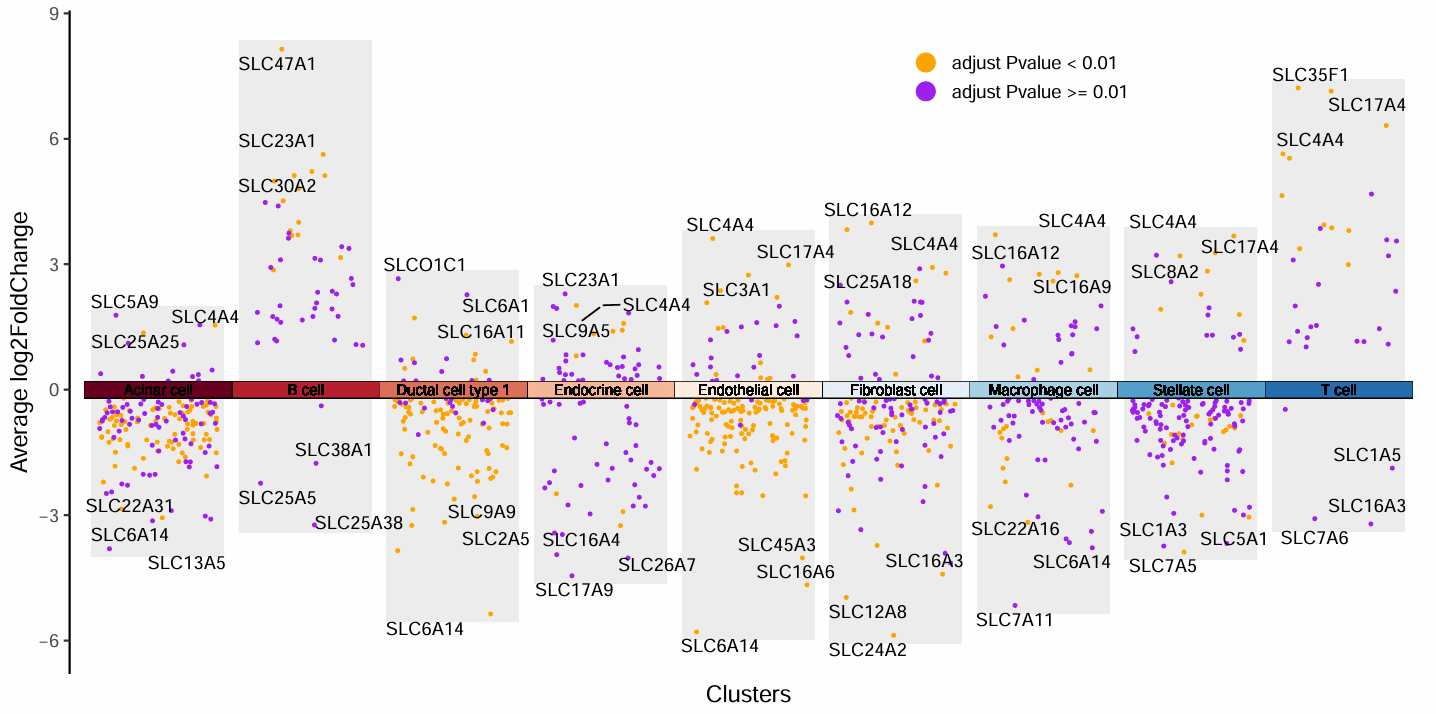


Fig S6. Differentially expressed SLC genes (DESGs) between tumor and normal samples in PAAD scRNA-seq dataset.


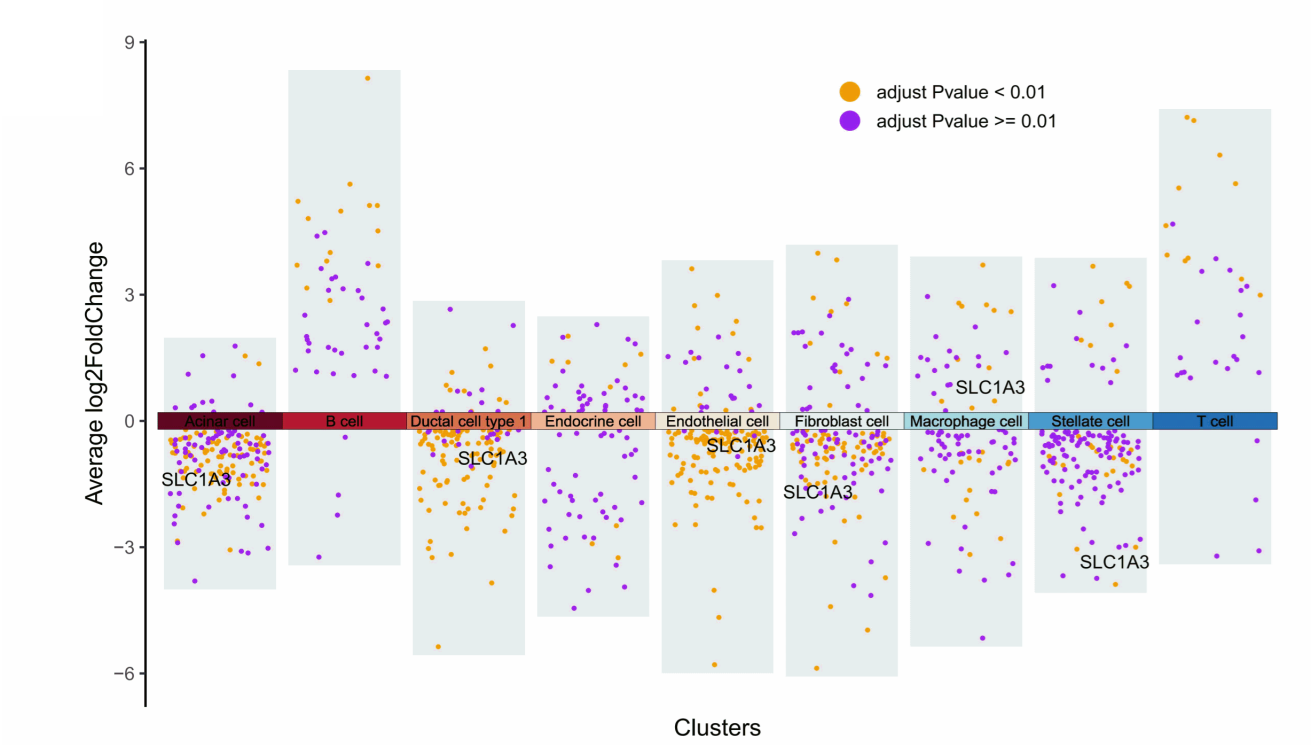


Fig S7. DESGs between tumor and normal cells in each cell type in PAAD scRNA-seq dataset. SLC1A3 was labeled.


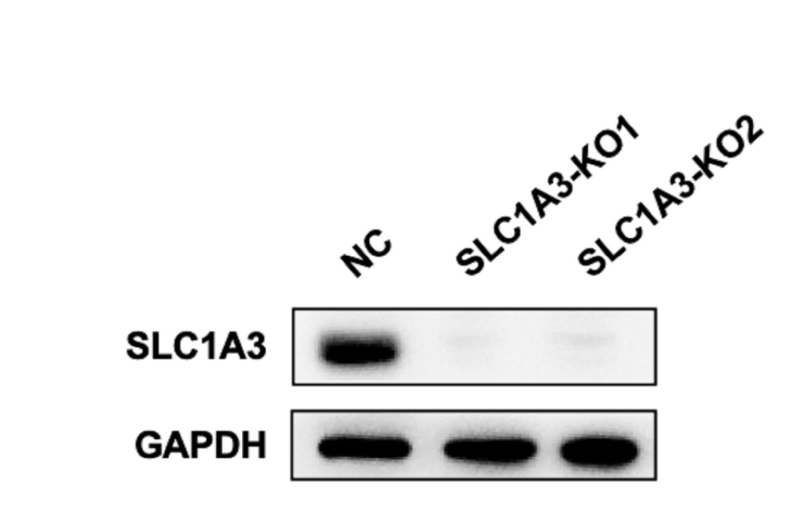


Fig S8. The SLC1A3 KO BxPC3 cell lines were confirmed respectively.


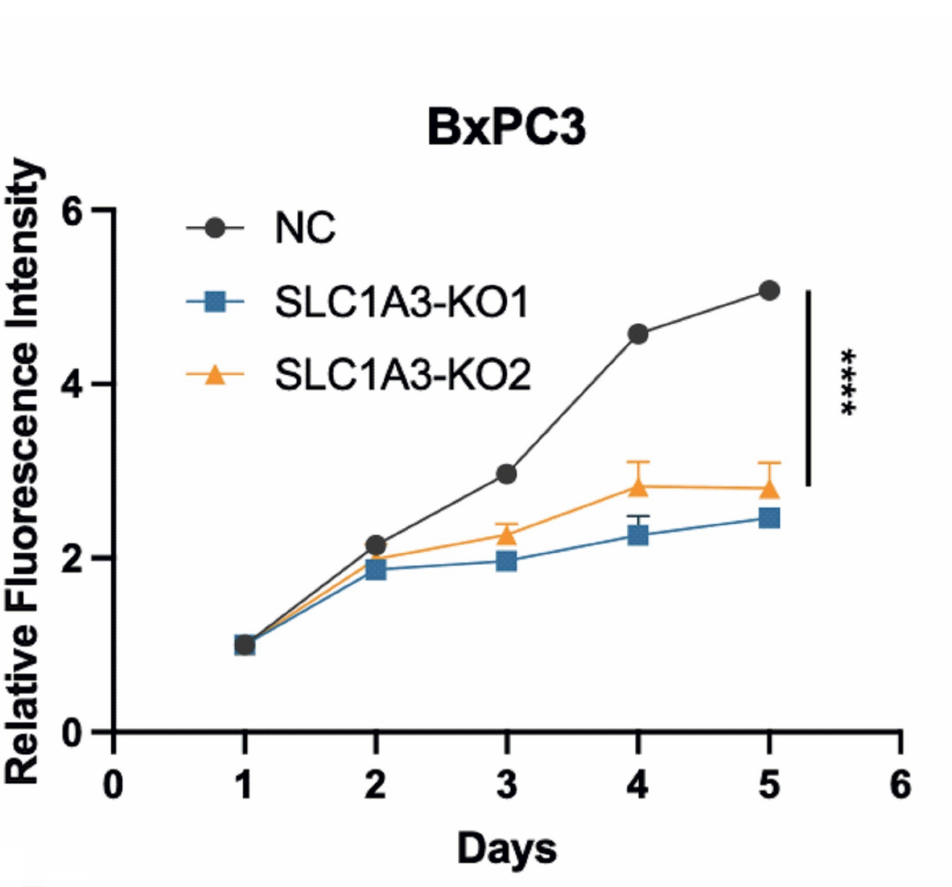


Fig S9. The SLC1A3 KO cell lines had relatively weak multiplication capacity compared with negative control.


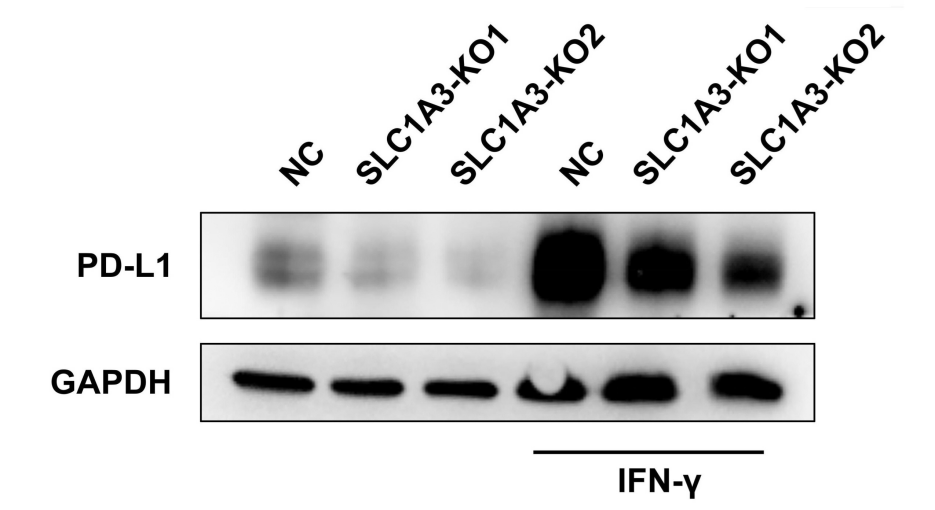


Fig S10. The expression of PD-L1 on the cell surface was down-regulated in both constitutive and induced via IFN--γ in BxPC3 cell lines knocked out by SLC1A3.


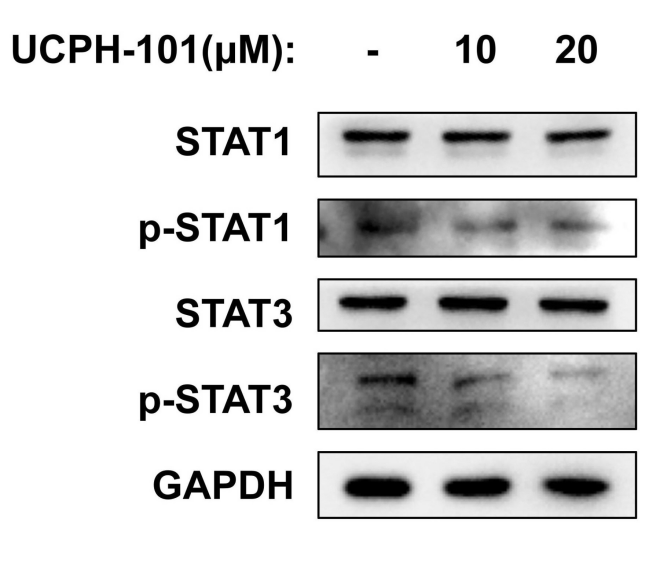


Fig S11. The SLC1A3 inhibitor UCPH-101 inhibits JAK/STAT signaling pathway activation.


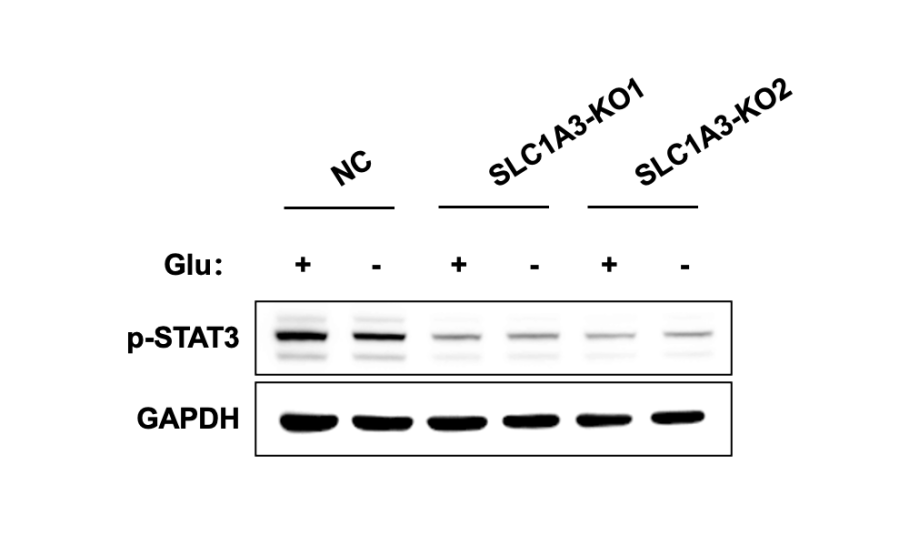


Fig S12. The JAK/STAT signaling pathway was inhibited in glutamate-deficient BxPC3 SLC1A3 KO cell culture.
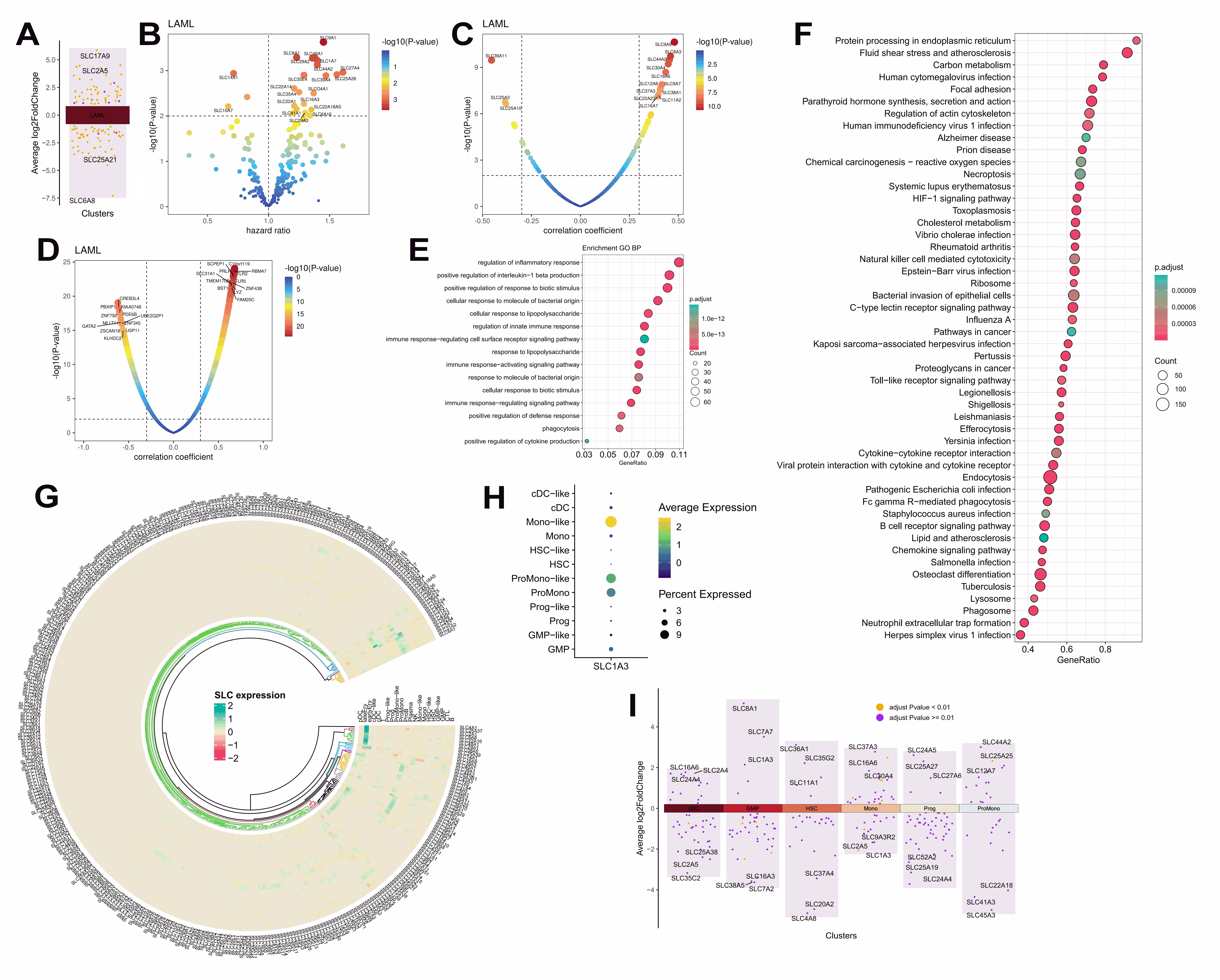


Fig S13. SLC1A3 is functional in tumor immunity in leukemia. (A) DESGs in LAML TCGA dataset (p<0.05, |log2FC|>1.0). (B) SRSGs in LAML. (C) PRSGs in AML. (D) Similar genes of SLC1A3 in LAML. (E) GO enrichment of PSGs of SLC1A3 in LAML. (F) GSEA of KEGG pathways for similar genes of SLC1A3 in LAML. (G) Expression profile of SLCs at single cell level. (H) expression of SLC1A3 in each cell type. (I) DESGs between malignant and normal cells in each cell type.
